# MP3: A Software Tool for the Prediction of Pathogenic Proteins in Genomic and Metagenomic Data

**DOI:** 10.1371/journal.pone.0093907

**Published:** 2014-04-15

**Authors:** Ankit Gupta, Rohan Kapil, Darshan B. Dhakan, Vineet K. Sharma

**Affiliations:** MetaInformatics Laboratory, Metagenomics and Systems Biology Group, Department of Biological Sciences, Indian Institute of Science Education and Research Bhopal, Madhya Pradesh, India; CSIR-Institute of Microbial Technology, India

## Abstract

The identification of virulent proteins in any de-novo sequenced genome is useful in estimating its pathogenic ability and understanding the mechanism of pathogenesis. Similarly, the identification of such proteins could be valuable in comparing the metagenome of healthy and diseased individuals and estimating the proportion of pathogenic species. However, the common challenge in both the above tasks is the identification of virulent proteins since a significant proportion of genomic and metagenomic proteins are novel and yet unannotated. The currently available tools which carry out the identification of virulent proteins provide limited accuracy and cannot be used on large datasets. Therefore, we have developed an MP3 standalone tool and web server for the prediction of pathogenic proteins in both genomic and metagenomic datasets. MP3 is developed using an integrated Support Vector Machine (SVM) and Hidden Markov Model (HMM) approach to carry out highly fast, sensitive and accurate prediction of pathogenic proteins. It displayed Sensitivity, Specificity, MCC and accuracy values of 92%, 100%, 0.92 and 96%, respectively, on blind dataset constructed using complete proteins. On the two metagenomic blind datasets (Blind A: 51–100 amino acids and Blind B: 30–50 amino acids), it displayed Sensitivity, Specificity, MCC and accuracy values of 82.39%, 97.86%, 0.80 and 89.32% for Blind A and 71.60%, 94.48%, 0.67 and 81.86% for Blind B, respectively. In addition, the performance of MP3 was validated on selected bacterial genomic and real metagenomic datasets. To our knowledge, MP3 is the only program that specializes in fast and accurate identification of partial pathogenic proteins predicted from short (100–150 bp) metagenomic reads and also performs exceptionally well on complete protein sequences. MP3 is publicly available at http://metagenomics.iiserb.ac.in/mp3/index.php.

## Introduction

The comparisons of completed bacterial genome sequences of closely related species have revealed significant genome variations between pathogenic and nonpathogenic bacteria [1]. One of the major differences between pathogenic and nonpathogenic bacteria is the presence of virulence-related genes in the former. These virulence genes could be present on bacterial plasmids or chromosomes, sometimes as pathogenicity islands, and are absent in nonpathogenic strains of the same or closely related species [2]. A well-known example is of closely related species belonging to *Shigella* and *Escherichia* genus, where the species belonging to the former are pathogenic and cause bacillary dysentery, whereas *Escherichia coli* (with the exception of some pathogenic strains) are commensals of the human gut microbiome [2], [3]. A recent study from *Chlamydiaceae* family indicated that porin proteins were significantly different in the outer membrane of chlamydial symbionts and pathogens [4]. Another study indicated that the differences in the capsular proteins in the pathogenic *Cryptococcus* species and environmental species influence their ability to cause virulence [5]. In eukaryotes, the sequence analysis of the pathogenic and nonpathogenic *Entamoeba histolytica* revealed significant evolutionary divergence and indicated that the pathogenic isolates are genetically distinct from the nonpathogenic isolates [6].

The mechanisms underlying pathogenesis are complex, diverse, species-specific, host-specific, and involve several processes including virulence, adhesion, invasion, secretion and drug resistance [7]. Due to this inherent complexity, the pathogenic species and the implicated proteins show considerable diversity and often exhibit insignificant similarity with the known proteins. Thus, it is difficult to predict such proteins by using homology-based methods such as BLAST [8] which is commonly used to assign function to a novel protein by alignment against a reference protein dataset [9], [10]. In addition, BLAST is relatively slow which further limits its usability on large genomic and metagenomic datasets. In this scenario, composition or profile-based approaches using Support Vector Machines (SVM) or Hidden Markov Model (HMM) could provide efficient and reliable alternatives.

There are two publicly available tools, VirulentPred and VICMpred, which have been developed to predict pathogenic proteins [9], [11]. VirulentPred is a SVM-based tool to predict virulent proteins in bacterial pathogens [9]. The SVM modules used in VirulentPred were trained using a combination of sequence features. A bilayer cascade SVM was developed in which the results from the first layer were cascaded to train and generate the second layer of SVM classifier. This bilayer cascade SVM provides an accuracy of 81.8%. Another method, VICMpred, is also developed using SVM-based approach. It predicts the major functions of Gram-negative bacterial proteins from their amino acid sequences and categorize them into virulence factors, information molecules, cellular process, and metabolism [11]. The features used in this method were calculated using PSI-BLAST similarity search, amino acid frequency, dipeptide frequency and tetrapeptide frequency. All these features were combined to form hybrid modules and it is able to achieve an overall accuracy of 70.75%. In addition to the above methods, two more computational methods have been developed to predict virulence factors in genomes. The first method predicts virulent proteins by integrating information for protein-protein interaction using STRING database and the information for biological pathways using the KEGG database, and then calculates a KEGG enrichment scores for the prediction [12]. This method provides a unique approach where KEGG pathways are used to predict virulence factors. However, the method was demonstrated only for three species and no publicly available tool is provided by the authors for using this approach. The second method, Virulent-GO, looks for informative gene ontology terms as features using a sequence-based approach for predicting bacterial virulent proteins [13]. However, no publicly available tool is provided for using this method. In addition, several databases such as Tox-Prot, VFDB, TVFac, ARGO, Islander, PRINTS virulence factors and SCORPION are also available which provide information on pathogenic proteins from both prokaryotes and eukaryotes [14]–[19]. MvirDB database has integrated information from several publicly available databases to construct a single useful resource containing protein sequences representing known toxins, virulence factors and antibiotic resistance genes [20]. In addition, it also contains sequences of pathogenic proteins reported in literature. Therefore, MvirDB can be used as a comprehensive resource to retrieve the information and sequences of pathogenic proteins.

Taken together, only a few tools are currently available for the prediction of pathogenic proteins and provide limited accuracy. Furthermore, they cannot be used on large-scale genomic or metagenomic datasets. Therefore, we have developed MP3 tool using an integrated SVM-HMM approach to provide improved efficiency and accuracy to predict pathogenic proteins in both genomic and metagenomic datasets. It is available as standalone tool as well as a publicly available web server.

## Materials and Methods

### Construction of Datasets

#### Positive and Negative Dataset

The performance of prediction methods primarily depends upon the quality of the training dataset which should be unambiguous and manually curated to achieve high accuracy in prediction. Therefore, in this study, the sequences of known virulence proteins were retrieved from MvirDB [20] which is a comprehensive microbial database of virulence factors, protein toxins and antibiotic resistance genes. Out of the total 64,711 proteins retrieved from MvirDB, 15,103 were selected using CD-HIT [21] such that no two sequences had 90% sequence identity. All the proteins annotated as hypothetical, putative, probable, possible or predicted were removed. This was followed by manual curation to remove the proteins with ambiguous annotations and to select the proteins of bacterial origin which are directly associated with any of the pathogenesis-related mechanisms including virulence, adhesion, invasion, secretion and drug resistance. The resulting positive dataset contained 1,708 protein sequences. To prepare the negative dataset, 10,411 protein sequences were retrieved from the Database of Essential Genes (DEG, version 8.0)[22]. To avoid overtraining of SVM, only one representative sequence was selected using CD-HIT among sequences having more than 90% sequence identity. From the 8,860 representative proteins selected after CD-HIT, the proteins annotated as hypothetical, putative, probable, possible, predicted were removed. This was followed by manual curation to remove the proteins having annotations similar to the proteins selected for constructing the positive dataset, and to remove those proteins which are known to play a direct role in pathogenesis. The proteins of the positive and negative dataset were further compared using CD-HIT-2D at 50% identity to check for the presence of any common proteins in the two datasets. The resulting negative dataset consisted of 5,815 proteins.

#### Blind Dataset

To assess the unbiased performance of the prediction method it was tested on blind dataset. The blind dataset was constructed using 100 negative proteins which were taken from the negative dataset and 100 positive proteins which were taken from the positive dataset and including 17 proteins from VFDB database. The resultant main blind dataset consisted of 200 proteins. The sequences in the blind dataset were never used before for the training purpose. After removing these proteins, the remaining positive and negative datasets contained 1,625 and 5,715 protein sequences, respectively, which were merged to create the main dataset consisting of 7,340 proteins.

#### Metagenomic Dataset

Two metagenomic sets (A and B) were constructed from the main dataset by randomly fragmenting proteins into 51–100 and 30–50 amino acids fragments, respectively, using in-house Perl scripts. The protein fragments of selected lengths corresponds to approximately 150–300 and 100–150 nucleotides, respectively, which mimics the lengths of real metagenomic reads generated from commonly used next-generation sequencers. Set A and B consisted of 48,715 and 83,761 fragments.

#### Metagenomic Blind Dataset

Two metagenomic blind datasets, BlindA (51–100 aa) and BlindB (30–50 aa) were constructed using the protein sequences of the Blind dataset. BlindA contained 2,604 protein fragments and BlindB contained 4,400 protein fragments. The fragments were generated using a similar methodology as described in the previous section.

#### Independent Genomic Datasets

Three independent sets were constructed to evaluate the performance of MP3. The first set consisted of 16 species of known pathogenic and nonpathogenic bacteria for which complete genome sequences are available at NCBI [23]. The second set consisted of three groups of proteins from the *Shigella flexineri* virulence plasmid as reported by Slogowski et al. [24]. The first group was composed of 18 proteins which are translocated by *Shigella* into host cells. The second group was composed of 20 proteins that are confined to the bacterium during infection (non-translocated). The third group was composed of three candidate translocated proteins based on the low GC content of their corresponding genes. Out of the total 38 proteins, 12 proteins were further shown to differentially (complete, intermediate or weak) inhibit yeast growth. In the third set, 200 proteins from a pathogenic *Mycobacterium tuberculosis* strain, *Mycobacterium tuberculosis Beijing NITR203* (known as Beijing strain), were selected from NCBI (ftp://ftp.ncbi.nlm.nih.gov/genomes/Bacteria/). Out of the 200 proteins, 100 are known and confirmed pathogenic proteins such as drug resistance proteins, MCE-family proteins and PE-PPE family proteins [25], [26]. The remaining 100 proteins are nonpathogenic and include polymerase proteins, ribosomal proteins and other proteins from essential genes which are not known to play any role in pathogenesis. MP3 was run on all the three genomic test datasets.

#### Independent Metagenomic Datasets

The performance of MP3 was also evaluated using real metagenomic datasets. The human gut microbiome datasets of a healthy European male individual (MH0050, Age 49) and a diseased European male individual (O2.UC-18, Age 48) were obtained from (ftp://public.genomics.org.cn/BGI/gutmeta/High_quality_reads/) [27]. The forward and reverse paired-end reads were assembled into 11,556,341 and 10,306,137 single reads for healthy and diseased datasets, respectively, using FLASH [28]. The MetaGeneMark [29] software was used for predicting ORFs which were analyzed using MP3 to identify the proportion of pathogenic proteins in the two datasets.

### Five-Fold Cross-Validation and Performance Evaluation

The performance of SVM module was evaluated using five-fold cross-validation by dividing the main dataset into five approximately equal parts. For the cross validation, four parts were used for training and the remaining part was used for testing. The process was repeated five times such that every part was used once for testing. The final performance was reported as the average of the values obtained after the five-fold cross-validation. The performance of SVM was examined using the following standard parameters.
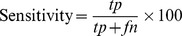





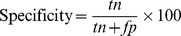





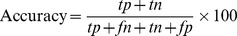





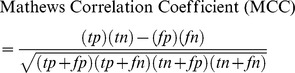



Where, tp (true positives) are the proteins which are known pathogenic and are predicted as pathogenic, and tn (true negatives) are the proteins which are known nonpathogenic and are predicted as nonpathogenic. Whereas, fp (false positives) are the proteins which are known nonpathogenic and are predicted as pathogenic, and fn (false negatives) are the proteins which are known pathogenic and are predicted as nonpathogenic.

### Calculation of Protein Features

#### Amino Acid and Dipeptide Composition

Amino acid composition and dipeptide composition of the protein sequences were evaluated as features for training SVM. While the amino acid composition only provides information about the percentage of each amino acid in the sequence, the dipeptide composition is more informative as it provides information about the fractions of amino acids as well as their local order in the form of a fixed-length vector which is used as the input for training SVM. Higher-order peptides, such as tripeptides and tetrapeptides, can also be used which can provide greater depth in the relative order of the amino acids in a protein but at the same time will increase the noise and redundancy. In addition, in case of small metagenomic ORFs, the higher-order peptides would be less informative. Thus, the AAC and dipeptide frequency have been used to evaluate the performance of SVM module. The amino acid composition and dipeptide composition of each protein was calculated using the formula given below.
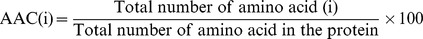
where, AAC(i) is the amino acid composition of the amino acid i, and amino acid (i) is one of the 20 amino acids.
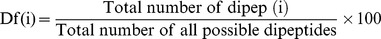



Where, Df(i) is the frequency of dipeptide i, and dipep(i) is one out of 400 dipeptides.

### Support Vector Machines (SVM)

SVM was implemented via SVM_Light package (http://svmlight.joachims.org/) [30] which provides options to choose a number of parameters and kernels (e.g. linear, polynomial, radial basis function and sigmoid) or any user-defined kernels. Among the available kernels, the polynomial kernel was selected since it provided better results for both genomic and metagenomic datasets as compared to other kernels (Figure S1, S5 and S6 in File S1). Therefore, polynomial kernel was used for all the SVM-based analysis carried out in this study and for constructing the SVM module for MP3.

### Implementation of Hidden Markov Models (HMM) Using Pfam Domains

HMM was implemented using HMMER3 software (http://hmmer.janelia.org/) [31]. The Pfam database (release 27.0) containing 14,831 families was retrieved (ftp://ftp.sanger.ac.uk/pub/databases/Pfam) [32].

#### Construction of MiniPfam Database

**Figure 1:**
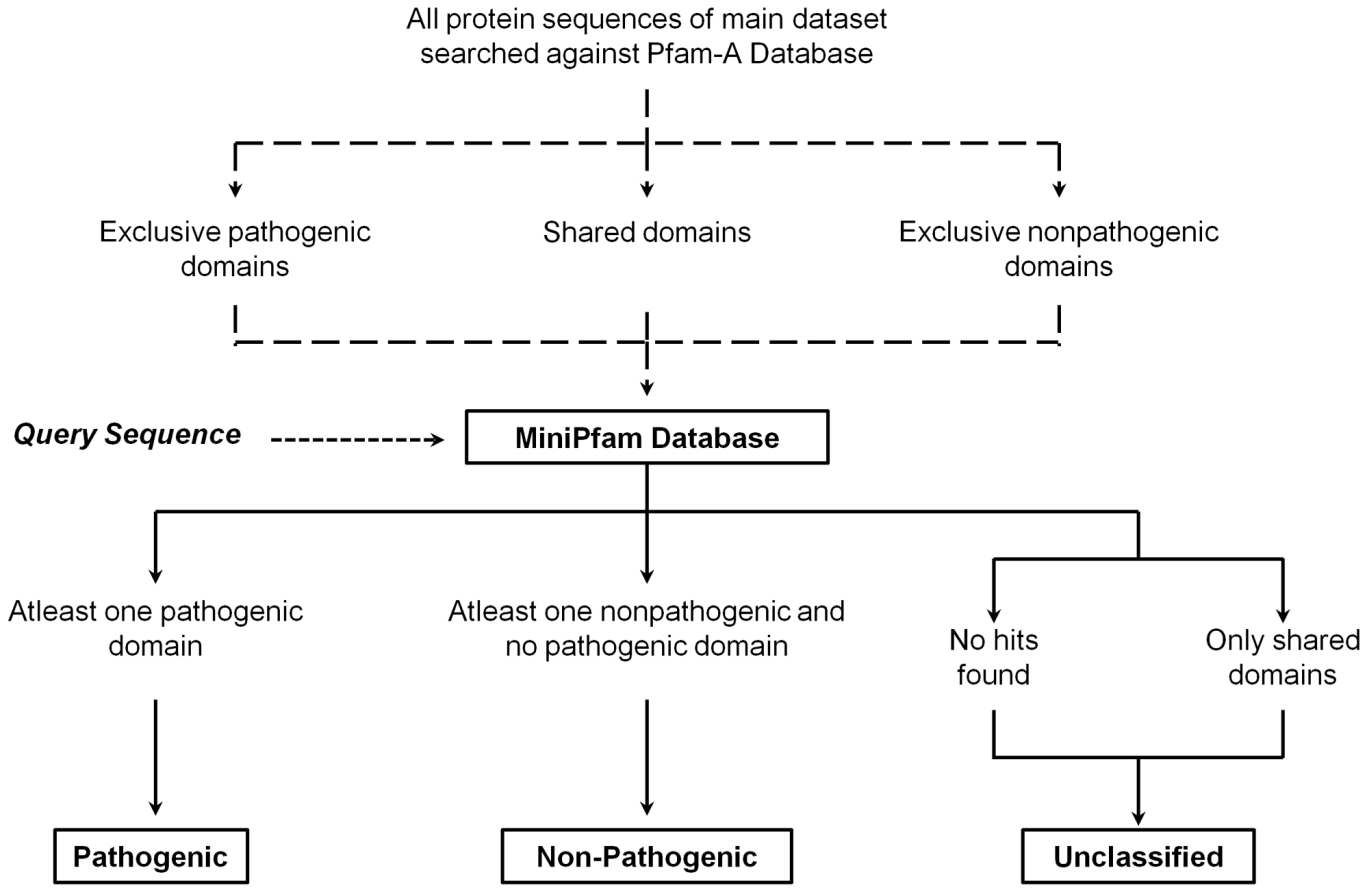
Steps used by HMM module for the prediction of pathogenic protein.

To construct a local database of pathogenic and nonpathogenic domains from the Pfam database, the protein sequences of the main dataset were searched against the Pfam database using HMMER at an e-value of 1e−5 (same e-value is used throughout the study for HMMER). The resulting domains were classified into three categories; (i) domains present only in pathogenic proteins (exclusive pathogenic), (ii) domains present only in nonpathogenic proteins (exclusive nonpathogenic) and, (iii) domains occurring in both pathogenic and nonpathogenic proteins (shared domains) (Figure 1). A total of 2,397 types of domains were found of which 498 domains were present exclusively in the pathogenic proteins, 1,636 domains were present exclusively in the nonpathogenic proteins, and 263 domains were present in both the pathogenic and nonpathogenic proteins. Using all the three types of domains, a local domain database ‘MiniPfam’ was constructed.

### Combined SVM-HMM Approach

The composition of proteins and the presence of functional domains can provide valuable insights about the function of a protein. Therefore, a combined approach using SVM (using dipeptide composition) and HMM (using Pfam domains) is used for the development of MP3 tool to achieve higher accuracy and sensitivity. Using the combined approach, all the protein sequences in the blind dataset were screened using both SVM and HMM modules. Among the two methods, SVM can classify a protein as either pathogenic or nonpathogenic, whereas, HMM can classify a protein as pathogenic, nonpathogenic or unclassified.

#### Other Databases

The Non-Redundant (NR) database (ftp://ftp.ncbi.nih.gov/blast/db/FASTA/, July 2013) was retrieved from NCBI [23] for the comparison of MP3 with BLAST.

## Results and Discussions

### Performance of SVM Modules on Genomic Datasets

The performance of SVM modules for genomic datasets was evaluated using amino acid composition and dipeptide composition as input features. The evaluation of the performance was carried out using five-fold cross validation. After trying all possible kernels and fine tuning the parameters, it was observed that RBF kernel (g = 0.01, c = 6) showed best performance for AAC based modules and polynomial kernel (d = 3, j = 4) showed best performance for dipeptide composition based modules as evident from the ROC plot (Figure S1 in File S1). The accuracies and MCC values of both the modules were almost similar at default threshold of zero; however, the sensitivity (76.12%) of dipeptide composition based module (Table 1) was much higher as compared to the sensitivity (63.20%) of AAC based modules (Table S1 and Figure S2 in File S1). Therefore, dipeptide composition with polynomial kernel was chosen as the input to the SVMs for all the further prediction on genomic datasets.

**Table 1:** Performance of SVM module on the main dataset.

Threshold	Sensitivity	Specificity	Accuracy	MCC
−1	95.08	50.34	59.89	0.38
−0.9	93.98	56.12	64.2	0.41
−0.8	93.43	61.6	68.39	0.45
−0.7	92.24	67.46	72.74	0.49
−0.6	89.65	72.71	76.33	0.52
−0.5	88.43	76.66	79.17	0.55
−0.4	86.56	80.27	81.61	0.58
−0.3	84.67	83.88	84.04	0.61
**−0.2**	**82.53**	**86.97**	**86.02**	**0.63**
−0.1	79.22	89.06	86.96	0.64
0	76.12	90.96	87.79	0.65
0.1	72.3	92.61	88.27	0.65
***0.2***	***69.66***	***93.94***	***88.75***	***0.66***
0.3	65.58	95.01	88.72	0.65
0.4	61.57	96.02	88.66	0.64
0.5	59.16	96.7	88.68	0.64
0.6	55.65	97.32	88.42	0.63
0.7	52.07	97.89	88.1	0.61
0.8	48.19	98.36	87.65	0.6
0.9	42.59	98.82	86.82	0.56
1	38.42	99.04	86.11	0.54

The point where sensitivity and specificity is roughly equal is highlighted in bold. The point of maximum MCC is highlighted in bold and italics.

The performance of SVM module with polynomial kernel and dipeptide composition as input is shown in Table 1. At the threshold of zero, the observed Sensitivity, Specificity, Accuracy and MCC were 76.12%, 90.96%, 87.79% and 0.65, respectively (Table 1). It is noticeable from Table 1 that the best possible combination of Sensitivity, Specificity, Accuracy and MCC was achieved at the threshold of −0.2 which are 82.53%, 86.97%, 86.02% and 0.63, respectively. Hence, −0.2 was selected as the default threshold for all further predictions by the SVM module.

The performance of SVM module was evaluated using blind dataset to determine the accuracy of predictions on unknown query sequences. At default (−0.2) threshold, a high accuracy (88%) of prediction was achieved on the blind dataset (Table 2).

**Table 2:** Performance of SVM, HMM and combined Modules on the Genomic Blind (Blind) and Metagenomic Blind datasets (BlindA and BlindB).

Method	Dataset	Sensitivity	Specificity	Accuracy	MCC
SVM	Blind	84	92	88	0.76
	BlindA	72.86	94.34	82.49	0.68
	BlindB	64.14	91.69	76.50	0.57
HMM	Blind (86.50%)*	100	97.02	98.27	0.97
	BlindA (52.60%)*	99.47	98.11	98.68	0.97
	BlindB (35.64%)*	78.17	99.03	89.24	0.80
Combined (MP3)	Blind	92	100	96	0.92
	BlindA	82.39	97.86	89.32	0.80
	BlindB	71.60	94.48	81.86	0.67

*The values in the brackets show the percentage prediction provided by HMM. The default threshold of −0.2 was used for SVM module and default e-value of 1e−5 was used for HMM module.

### Performance of SVM Modules on Metagenomic Datasets

Using a similar methodology as used above for the genomic datasets, the best kernel and parameters for SVM were selected for the two metagenomic datasets (set A and set B). For both the metagenomic datasets, the performance of SVM modules using dipeptide composition (Table 3) was better as compared to SVM modules using AAC (Table S2 in File S1) as the input (Figure S3 and S4 in File S1). Hence, using dipeptide frequency as input, polynomial kernel (with d = 4, C = 51 for set A and d = 3, C = 3 for set B), which showed the best performance among all available kernels (Figure S5 and S6 in File S1), was selected for all further predictions by SVM on metagenomic datasets. The accuracies of 93.25% for set A and 91.48% for set B were achieved at zero threshold (Table 3). However, the best combination of Sensitivity, Specificity, Accuracy and MCC for dataset A and B were achieved at the default threshold of −0.2 (Table 3). The performance of SVM module was further evaluated using BlindA and BlindB datasets and accuracies of 82.49% and 76.5%, respectively, were achieved (Table 2).

**Table 3:** Performance of SVM module on metagenomic datasets.

Threshold	Sensitivity		Specificity		Accuracy		MCC	
	A	B	A	B	A	B	A	B
−1	99.32	98.41	35.87	35.47	52.30	51.58	0.35	0.33
−0.9	98.81	97.61	48.67	47.32	61.65	60.19	0.43	0.41
−0.8	98.10	96.53	60.94	58.58	70.56	68.29	0.52	0.48
−0.7	97.05	95.23	71.56	68.78	78.16	75.55	0.60	0.56
−0.6	95.89	93.37	79.90	77.20	84.04	81.34	0.68	0.63
−0.5	94.05	90.98	86.26	83.89	88.28	85.70	0.74	0.68
−0.4	91.83	88.19	91.00	88.85	91.22	88.69	0.79	0.73
−0.3	89.35	84.91	94.37	92.59	93.07	90.62	0.82	0.76
**−** ***0.2***	***85.59***	***81.41***	***96.64***	***95.06***	***93.78***	***91.57***	***0.84***	***0.78***
−0.1	81.85	77.57	97.92	96.71	93.76	91.81	0.83	0.78
0	77.49	73.09	98.75	97.80	93.25	91.48	0.82	0.77
0.1	72.72	68.52	99.18	98.53	92.33	90.85	0.80	0.75
0.2	67.60	63.46	99.45	99.06	91.20	89.95	0.77	0.73
0.3	62.30	58.51	99.59	99.34	89.94	88.89	0.73	0.70
0.4	57.26	52.82	99.73	99.54	88.74	87.59	0.70	0.66
0.5	51.58	47.25	99.82	99.69	87.33	86.28	0.66	0.62
0.6	45.38	41.17	99.85	99.78	85.75	84.78	0.61	0.58
0.7	39.59	35.47	99.88	99.84	84.27	83.37	0.57	0.53
0.8	32.97	29.28	99.91	99.89	82.58	81.82	0.51	0.48
0.9	27.32	23.74	99.94	99.92	81.14	80.43	0.46	0.43
1	21.99	17.65	99.96	99.94	79.77	78.88	0.41	0.37

‘A’ refers to metagenomic Set A, and ‘B’ refers to metagenomic Set B. The point where sensitivity and specificity is roughly equal, and MCC is maximum is highlighted in bold and italics.

### Performance Evaluation of HMM Module

The performance of HMM module was evaluated on the main dataset by searching each protein against the MiniPfam database by HMMER using an e-value of 1e−5. A protein is classified as ‘pathogenic’ if it contains at least one pathogenic domain (Figure 1). Similarly, a protein is classified as ‘nonpathogenic’ if it does not contain any pathogenic domain and contains at least one nonpathogenic domain. The remaining proteins containing only shared domains or for which no hits are found are categorized in the ‘unclassified’ category. Using this approach on the main dataset, HMM module could make predictions on 1,167 (1153 correct and 14 incorrect) proteins in the positive dataset and 5,405 (5,375 correct and 30 incorrect) proteins in the negative dataset with high Sensitivity (98.80%), Specificity (99.44%), Accuracy (99.33%) and MCC (0.98) (Table S3 in File S1). The residual 768 proteins remained unclassified. HMM module showed an accuracy of 98.27% on the Blind dataset (Table 2). For the positive blind dataset, it correctly predicted 72 proteins out of 100 proteins with 3 incorrect predictions, and 25 remained as unclassified (Table S3 in File S1). For the negative blind dataset, 98 out of 100 proteins were predicted correctly and two proteins remained as unclassified (Table S3 in File S1).

### Performance Evaluation of HMM Module on Metagenomic Dataset

HMM module showed exceptionally high accuracies on the metagenomic datasets, however, it could classify a limited proportion of metagenomic protein fragments. A plausible reason is that a metagenomic read (length 100–400 bp) can originate from any part of a gene and may contain only a partial fragment of that gene. In many cases small protein fragments of 30–100 amino acids length may not contain any domain or contain an insignificant part of the domain, and hence, does not find a match using the HMM module. For Set A, 60.75% of the protein fragments could be classified with high Sensitivity (99.25%), Specificity (99.78%), Accuracy (99.69%) and MCC (0.99). Similarly, for Set B, HMM module could classify 41.40% protein fragments as either pathogenic or nonpathogenic with high Sensitivity (99.68%), Specificity (99.93%), Accuracy (99.89%) and MCC (0.99). The detailed information is provided in Table S4 and S5 in File S1.

The performance of HMM module was further evaluated on the metagenomic blind datasets (BlindA and BlindB). In BlindA set, 52.6% of the total protein fragments could be classified with an accuracy of 98.68%. Similarly, 35.64% of the total protein fragments of BlindB could be classified with an accuracy of 89.24% (Table 2).

### Combined SVM-HMM Approach to Develop MP3

**Figure 2:**
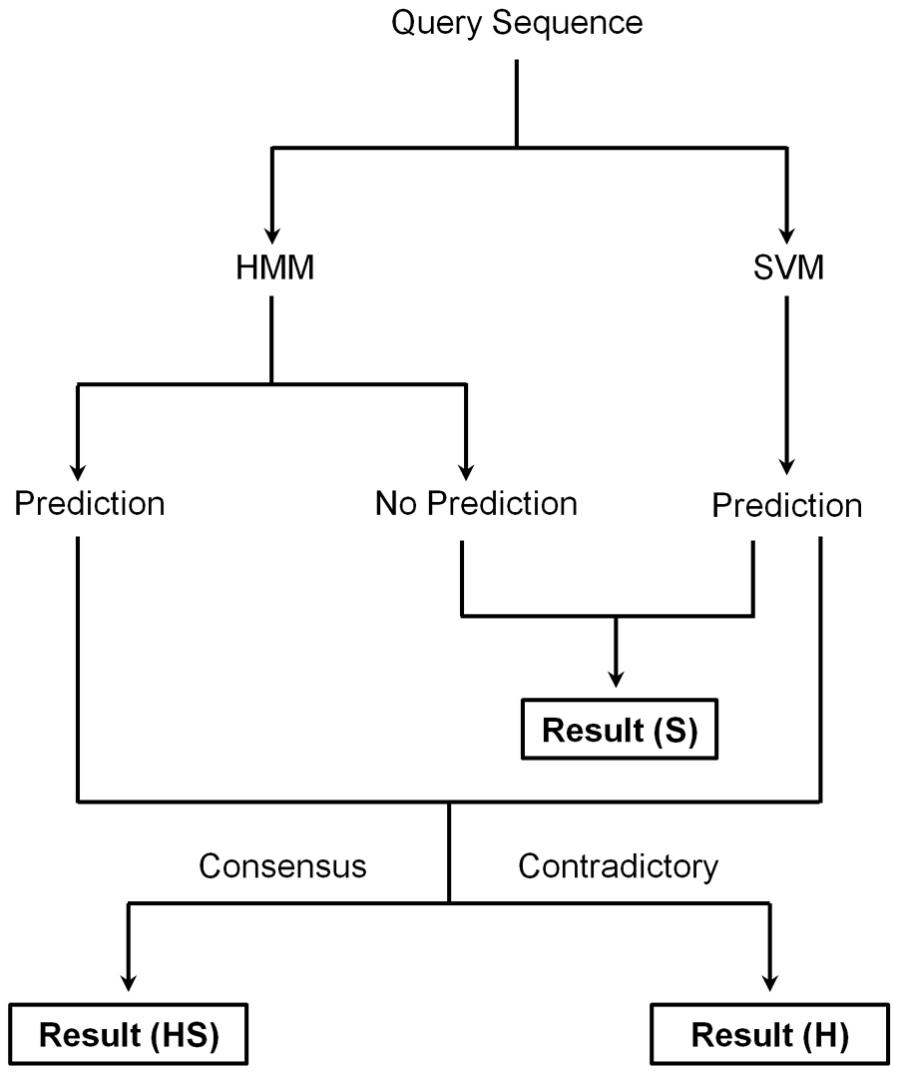
Prediction of pathogenic or nonpathogenic proteins using MP3. HS: predictions from both HMM and SVM are in consensus, H: prediction is based only on HMM module, S: prediction is based only on SVM module.

To improve the sensitivity and accuracy of the predictions, a SVM-HMM combined approach was implemented to develop the MP3 tool. The criteria used to carry out the assignments are shown in Figure 2. It is to be noted that for the cases where SVM and HMM modules made different predictions, the predictions of HMM were considered. The reason for giving preference to HMM predictions over SVM predictions is because for both genomic and metagenomic datasets, though the number of predictions made by HMM was lesser as compared to SVM, HMM showed higher sensitivity, specificity and accuracy as compared to SVM. The prediction made by MP3 is assigned with ‘HS’ if the predictions from both HMM and SVM are in consensus. HS labelled predictions can be considered highly accurate. ‘H’ or ‘S’ are assigned when the prediction result is based either on HMM or SVM module, respectively (Figure 2).

Following the above approach, performance of MP3 was tested on genomic and metagenomic blind datasets. MP3 showed an accuracy of 96% in case of genomic blind dataset and an accuracy of 89.32% and 81.86% in case of metagenomic blind datasets BlindA and BlindB, respectively (Table 2). The detailed comparison of MP3 (integrated SVM-HMM) with SVM and HMM for the genomic and metagenomic blind datasets is shown in Table S6 in File S1.

### Performance of MP3 on Genomic and Metagenomic Independent Datasets

The performance of MP3 was tested on publicly available genomic and metagenomic datasets. On the first independent dataset consisting of 16 pathogenic and nonpathogenic bacterial genomes, the percentage of pathogenic proteins predicted by MP3 is higher in the pathogenic genomes as compared to the nonpathogenic genomes (Table 4). MP3 predicted 20.4%, 23.7% and 30.28% of the total proteins as pathogenic in the case of pathogenic *Mycobacterium* species, *Mycobacterium leprae TN*, *Mycobacterium tuberculosis str. Beijing NITR203* and *Mycobacterium tuberculosis H37Rv*, respectively. Interestingly, MP3 predicted 224, 447 and 198 unannotated proteins as pathogenic in *Mycobacterium leprae TN* and *Mycobacterium tuberculosis str. Beijing NITR203* and *Mycobacterium tuberculosis H37Rv*, respectively (Text S1 in File S1). Given the highly accurate performance of MP3 on the test dataset derived from *Mycobacterium tuberculosis str. Beijing NITR203*, the unannotated proteins predicted as pathogenic in the three pathogenic *Mycobacterium* species provide new leads for experimental validations to confirm their role in the pathogenesis of *Mycobacterium*.

**Table 4:** Performance of MP3 on known bacterial genomes.

Genome	Type	Number of Pathogenic Proteins (%)
*Arcobacter nitrofigilis DSM 7299*	N	249 (13.2)
*Bacillus anthracis str. A0248*	P	1,052 (20.8)
*Bacillus subtilis subsp. subtilis str. 168*	N	778 (18.6)
*Escherichia coli O157:H7 str. EC4115*	P	1,291(24.3)
*Escherichia coli str. K-12 substr. DH10B*	N	885 (21.5)
*Lactobacillus fermentum F-6*	N	261 (13.0)
*Mycobacterium Tuberculosis H37Rv*	P	938(30.28)
*Mycobacterium leprae TN*	P	328 (20.4)
*Mycobacterium smegmatis str. MC2 155*	N	485 (10.9)
*Mycobacterium tuberculosis str. Beijing/NITR203*	P	973 (23.7)
*Neisseria lactamica 020-06*	N	310 (15.7)
*Neisseria meningitides 053442*	P	330 (16.3)
*Pseudomonas aeruginosa B136-33*	P	1,527 (26.2)
*Pseudomonas putida BIRD-1*	N	1,153 (23.24)
*Shigella flexineri 2a str. 2457T*	P	759 (18.7)
*Vibrio cholerae IEC224*	P	474 (18.0)

The threshold of 0.2 was used to achieve high specificity for the above analysis by SVM module.

Type indicates pathogenicity of the bacteria indicated by ‘N’ for nonpathogenic and ‘P’ for pathogenic bacteria.

To compare the performance of MP3 with BLAST, the 869 hypothetical proteins from all selected pathogenic *Mycobacterium* species which were predicted as pathogenic by MP3 were searched against the NCBI-NR database using BLASTP. The best hit was selected using the default e-value of 10. Out of the total 869 hypothetical proteins, functional annotations could be found for only 43 proteins and 44 proteins were found annotated with only general functions. To specifically classify these proteins into pathogenic or non-pathogenic classes, manual efforts are needed to go through their annotations and interpret their role as pathogenic or nonpathogenic protein. Therefore, MP3 could serve as a useful tool to classify the hypothetical proteins as pathogenic or nonpathogenic. In addition, the performance of MP3 was up to 2000 times faster than BLAST for a sample set containing 2,000 proteins (Table S7 in File S1).

For the nonpathogenic *Mycobacterium smegmatis str. MC2 155*, 10% of the total proteins were predicted as pathogenic. Though, *Mycobacteirum smegmatis* is a nonpathogenic species but its genome also contains a number of known pathogenic proteins such as PE-PPE family proteins, MCE family proteins, drug resistance proteins, and enzymes. Therefore, such proteins were predicted as pathogenic in *M. smegmatis* by MP3. However, the total number of pathogenic proteins in pathogenic species of *Mycobacterium*, i.e. *Mycobacterium tuberculosis*, is much higher as compared to its nonpathogenic species, i.e. *Mycobacterium smegmatis*. In addition, a small proportion of proteins were predicted as pathogenic in other nonpathogenic genomes. The plausible reason could be that the mechanisms of pathogenesis involve several proteins which are either directly or indirectly involved in the process. Therefore, it is expected that some of the associated pathogenic proteins which may not be directly involved in pathogenesis, such as enzymes, flagellar proteins, fimbrial proteins, membrane proteins, transport proteins, or secretory proteins, may be present in both pathogenic and nonpathogenic genomes. Since such proteins are shared between the pathogenic and nonpathogenic species, they were considered in the positive dataset for the training of SVM and HMM and will be predicted as pathogenic by MP3. However, it is noticeable that MP3 predicted much higher number of pathogenic proteins in the pathogenic genomes.

In the case of second independent dataset consisting of genes present on virulence plasmid of *Shigella*, 17 out of the 18 proteins from group I (translocated proteins) and 6 out of 20 proteins from group II (non-translocated proteins) were predicted as pathogenic (Table S8 in File S1). These predictions concur with the results shown in the study by Slogowski et al. where they observed that the expression of translocated proteins resulted in greater growth inhibition than non-translocated proteins. It was also shown in the above study that 12 out of the total 38 proteins could differentially (complete, intermediate and weak) inhibit yeast growth. MP3 was able to correctly predict 9 of these 12 as definite virulence proteins (Table 5). The three misclassified proteins were plasmid segregation proteins (mvpT and parA) and a protein of unknown function (OspD3). Among these, mvpT is predicted as nonpathogenic but it was assigned with ‘H’, i.e, the prediction is based only on the results of HMM, and the prediction of SVM and HMM were not in consensus. The protein parA was assigned with ‘HS’ indicating that it is predicted as nonpathogenic by MP3 with high confidence. Though mvpT and parA proteins were shown as a pathogenic protein by Slogowski et al., their function as plasmid segregation proteins can be considered as a general function which is present in both pathogenic and nonpathogenic genomes. Thus, these proteins were classified as nonpathogenic by MP3. The third protein OspD3 is of unknown function and thus, the possible reason for its classification as a nonpathogenic protein is not clear. These results further support the accuracy of MP3.

**Table 5:** Performance of MP3 on second independent dataset consisting of proteins from virulence plasmid of *Shigella flexineri*.

Protein	Secreted	Translocated	Function	Inhibition	MP3 prediction	Tag
icsB	Y	Y	Inhibits autophagy	Complete	Pathogenic	S
IpgGB2	N	N	G-protein mimic	Complete	Pathogenic	S
IpgD	Y	y	Inositol phosphate phosphatase	Complete	Pathogenic	S
VirA	Y	Y	Microtubule-severing activity	Complete	Pathogenic	S
mvpT	N	N	Toxin- plasmid segregation	Complete	Non-Pathogenic	H
IpaJ	N	N	Unknown	Complete	Pathogenic	S
IpgGB1	Y	y	G-protein mimic	Intermediate	Pathogenic	S
OspC1	Y	N	Unknown	Intermediate	Pathogenic	S
OspD3	N	N	Unknown	Intermediate	Non-Pathogenic	S
OspF	Y	Y	MAPK phosphothreonine	Intermediate	Pathogenic	S
parA	N	N	Plasmid segregation	Intermediate	Non-Pathogenic	HS
OspB	Y	N	Unknown	Weak	Pathogenic	S

Y: refers to Yes; N: refers to No. The complete results for all the 38 proteins are provided in Table S8 in File S1.

The performance of combined approach was also tested on publicly available metagenomic datasets of one healthy and one diseased European male individual containing paired-end reads generated by Illumina GA [27]. A total of 8,026,105 and 6,952,195 ORFs (length between 30–50 amino acids) were predicted in healthy and diseased datasets using MetaGeneMark [29]. MP3 was run on the ORFs predicted in the two datasets and it took ∼180 CPU hours (Intel Xeon 2.4 GhZ CPU) to carry out the assignment which is really reasonable considering the size of input data. MP3 predicted 16.51% and 19.37% proteins as pathogenic in healthy and diseased individuals, respectively. These results validate the efficiency and capability of MP3 in predicting pathogenic proteins in the metagenomic datasets.

### Comparison with Other Web Servers

The performance of MP3 was compared with publicly available VirulentPred web server which can predict virulent proteins in genomic datasets. On blind dataset constructed in this study, the Sensitivity (92%), Specificity (100%), Accuracy (96%) and MCC (0.92) achieved by MP3 is much higher than the Sensitivity (61.24%), Specificity (70.42%), Accuracy (64.5%) and MCC (0.30) obtained by VirulentPred (Table S9 in File S1). On the independent dataset provided by VirulentPred, MP3 exhibited an accuracy of 90% whereas VirulentPred showed an accuracy of 85%. The higher accuracy shown by MP3 on an independent dataset used for the evaluation of VirulentPred attests to the accuracy of MP3 on any unknown dataset. The other publicly available tool VICMpred can accept only a single sequence at a time and therefore could not be used for the comparison.

The performance of MP3 was also compared with VirulentPred on third independent dataset consisting of 200 known pathogenic and nonpathogenic proteins derived from pathogenic *Mycobacterium tuberculosis Beijing NITR203* strain. The Sensitivity (97%), Specificity (97%), Accuracy (97%) and MCC (0.94) achieved by MP3 is much higher than the Sensitivity (81%), Specificity (34%), Accuracy (57.5%) and MCC (0.16) obtained by VirulentPred. These results indicate that MP3 displays much better performance than the other available methods.

### Description of Web Server

MP3 web server and standalone program was developed using the combined SVM-HMM approach. The web server can be used as an online resource to identify the pathogenic proteins in both genomic and metagenomic datasets. On the Applications page, two options, namely ‘Genomic’ and ‘Metagenomic’ are provided to analyze the complete (genomic) proteins or partial (metagenomic) proteins. User can upload a File containing the protein sequences in FASTA format. Using the ‘Threshold’ option, a threshold (cut-off used by SVM module) to classify the input proteins as pathogenic or nonpathogenic can be specified, or a default threshold will be used in case no threshold value is provided. For the ‘Metagenomic’ option, the estimated length of protein sequences should be specified as less than or greater than 50 amino acids to select an appropriate SVM model which will be used by the SVM module of the MP3 tool. On submission of a query, a ‘Job ID’ page is displayed showing the link to the ‘Results’ page and an email is sent to the user. The ‘Results’ page displays the summary of the results and links to download all the results files. The MP3 web server is freely accessible at http://metagenomics.iiserb.ac.in/mp3/index.php. The standalone version of MP3 and detailed installation instructions are available at http://metagenomics.iiserb.ac.in/mp3/download.php.

## Conclusion

The combined SVM-HMM approach implemented as ‘MP3’ tool can carry out fast, sensitive and accurate prediction of virulent proteins in both metagenomic and genomic datasets. MP3 specializes in the identification of fragments of virulent proteins which are common in metagenomic data and can be used to compare the proportion of pathogenic proteins in a healthy and diseased sample without the use of time-consuming homology-based alignment. In addition, it also carries out the prediction of virulent proteins in complete genomes with greater accuracy, sensitivity and specificity as compared to other publicly available methods. At present, to the best of our knowledge, MP3 is the only program and web server which can predict pathogenic proteins in metagenomic datasets and in addition, can also predict pathogenic proteins in genomic datasets with such high accuracy and sensitivity. The MP3 standalone program and web server will serve as a valuable tool for biologists in predicting pathogenic proteins in both genomic and metagenomic datasets.

## Supporting Information

File S1 **Table S1**, Performance of SVM modules on genomic dataset using Amino acid composition as input (Learning parameters: t 2 g 0.01 c 6). The point where sensitivity and specificity is roughly equal is highlighted in bold. The point of maximum MCC is highlighted in bold and italics. **Table S2**, Performance of SVM module on the metagenomic datasets using amino acid composition as input (parameters for set B: t 2 g 0.002 c 6 and for set A - t 2 g 0.01 c 11). The point of maximum MCC is highlighted in bold and in Red color for Set A. The point of maximum MCC is highlighted in bold and in Blue color for Set B. **Table S3**, Performance of HMM modules on main and blind genomic dataset. The detailed data for the predictions made by HMM module are provided in the above table. Out of 7,340 proteins of the main dataset, HMM module could classify 89.5% of the proteins with high Sensitivity (98.80%), Specificity (99.44%), Accuracy (99.33%) and MCC (0.98) values. Similarly, out of 200 proteins of the blind dataset, HMM module could classify 86.5% of the proteins with an accuracy of 98.26%. **Table S4**, Performance of HMM modules on metagenomic Set A. The detailed data for the predictions made by HMM module is provided in the above table. Out of 48,715 protein fragments of the main dataset, HMM module could classify 60.75% of the proteins with high Sensitivity (99.25%), Specificity (99.78%), Accuracy (99.69%) and MCC (0.99) values. Similarly, out of 2,604 protein fragments of the blind dataset, HMM module could classify 52.60% of the protein fragments with an accuracy of 98.68%. **Table S5**, Performance of HMM modules on metagenomic Set B. The detailed data for the predictions made by HMM module is provided in the above table. Out of 83,761 protein fragments of the main dataset, HMM module could classify 41.40% of the proteins with high Sensitivity (99.68%), Specificity (99.93%), Accuracy (99.89%) and MCC (0.99) values. Similarly, out of 4,400 protein fragments of the blind dataset, HMM module could classify 35.64% of the protein fragments with an accuracy of 89.24%. **Table S6**, Comparison of the performance of SVM-HMM Hybrid approach with HMM and SVM modules. P – Positive dataset, N - Negative dataset. Default threshold for SVM module was −0.2 and default e-value of 1e-5 was used for HMM module. Since, the generation of number of fragments depends on the length of the proteins, therefore, there are unequal number of fragments in the positive and negative datasets for Blind A and B. In case of HMM, the number of correctly and incorrectly predicted proteins does not sum up to the total number because HMM does not make prediction on all the proteins. **Table S7**, Comparison of time taken by MP3 and BLAST. **Table S8**, Results of MP3 on the three groups of proteins from the *Shigella flexineri* virulence plasmid. **Table S9**, Comparison of MP3 and VirulentPred on different test datasets. The default threshold of −0.2 was used for SVM module and default e-value of 1e-5 was used for HMM module. **Figure S1**, Comparison of performance of different kernels of SVM on genomic dataset shown by ROC plot. The area under the ROC curve (AUC) for polynomial, RBF and linear kernel is 0.91, 0.91 and 0.86, respectively. Though, RBF and polynomial kernel have same AUC, however, the sensitivity value at zero threshold was higher in the polynomial kernel (76.12%) as compared to RBF kernel (73.39%). Hence, polynomial kernel was selected for the prediction by SVM modules. **Figure S2**, Comparison of performance of SVM modules using Amino Acid Composition (AAC) and dipeptide frequency as feature input for genomic dataset shown by ROC plot. The Area under the ROC for SVM modules with dipeptide composition and amino acid composition are 0.91 and 0.90, respectively. The Area under the ROC curve is almost same in both the modules, however, the sensitivity value of AAC (63.20%) based module was much lower as compared to dipeptide composition based module (76.12%). Hence dipeptide composition modules were selected over AAC based modules. **Figure S3**, Comparison of performance of SVM modules using Amino Acid Composition (AAC) and dipeptide frequency as feature input for metagenomic dataset A shown by ROC plot. The performance of dipeptide composition based module was far much better as compared to AAC composition based module as apparent from the Figure. The area under the ROC curve for AAC module and dipeptide composition based module were 0.83 and 0.97 respectively. Hence, dipeptide composition was selected as feature input for the SVM modules constructed for metagenomic dataset A. **Figure S4**, Performance comparison of SVM modules using Amino Acid Composition (AAC) and dipeptide frequency as feature input for metagenomic dataset B shown by ROC plot. The performance of dipeptide composition based module was far much better as compared to AAC composition based module as apparent from the Figure. The area under the ROC curve for AAC module and dipeptide composition based module were 0.84 and 0.95 respectively. Hence, dipeptide composition was selected as feature input for the SVM modules constructed for metagenomic dataset B. **Figure S5**, Performance comparison of different kernels of SVM on metagenomic dataset A (50 -100 aa) shown by ROC plot. The area under the ROC curve for polynomial, RBF and linear kernel is 0.97, 0.97 and 0.80 respectively. In this case, polynomial and RBF kernels have similar performance for metagenomic dataset A. However, in all other cases, polynomial kernel showed better results, therefore, the polynomial kernel was selected as the default kernel for all the analysis using SVM. **Figure S6**, Performance comparison of different kernels of SVM on metagenomic dataset B (30–50 aa) shown by ROC plot. The area under the ROC curve for polynomial, RBF and linear kernel is 0.95, 0.91 and 0.75 respectively. As it is clearly seen the polynomial kernel is performing better then both the other kernels, hence, polynomial kernel was selected for the SVM modules constructed for metagenomic dataset B. **Text S1**, The GI numbers of all the hypothetical proteins of the three pathogenic strains of *Mycobacterium* which were predicted as pathogenic by MP3 are given below.
(DOCX)
