# Supplementary material for: MP3: A Software Tool for the Prediction of Pathogenic Proteins in Genomic and Metagenomic Data
Source: PLoS One. 2014 Apr 15;9(4):e93907. doi: 10.1371/journal.pone.0093907 (PMC3988012; doi:10.1371/journal.pone.0093907)
Supplement: File S1 — Table S1, Performance of SVM modules on genomic dataset using Amino acid composition as input (Learning parameters: t 2 g 0.01 c 6). The point where sensitivity and specificity is roughly equal is highlighted in bold. The point of maximum MCC is highlighted in bold and italics. Table S2, Performance of SVM module on the metagenomic datasets using amino acid composition as input (parameters for set B: t 2 g 0.002 c 6 and for set A - t 2 g 0.01 c 11). The point of maximum MCC is highlighted in bold and in Red color for Set A. The point of maximum MCC is highlighted in bold and in Blue color for Set B. Table S3, Performance of HMM modules on main and blind genomic dataset. The detailed data for the predictions made by HMM module are provided in the above table. Out of 7,340 proteins of the main dataset, HMM module could classify 89.5% of the proteins with high Sensitivity (98.80%), Specificity (99.44%), Accuracy (99.33%) and MCC (0.98) values. Similarly, out of 200 proteins of the blind dataset, HMM module could classify 86.5% of the proteins with an accuracy of 98.26%. Table S4, Performance of HMM modules on metagenomic Set A. The detailed data for the predictions made by HMM module is provided in the above table. Out of 48,715 protein fragments of the main dataset, HMM module could classify 60.75% of the proteins with high Sensitivity (99.25%), Specificity (99.78%), Accuracy (99.69%) and MCC (0.99) values. Similarly, out of 2,604 protein fragments of the blind dataset, HMM module could classify 52.60% of the protein fragments with an accuracy of 98.68%. Table S5, Performance of HMM modules on metagenomic Set B. The detailed data for the predictions made by HMM module is provided in the above table. Out of 83,761 protein fragments of the main dataset, HMM module could classify 41.40% of the proteins with high Sensitivity (99.68%), Specificity (99.93%), Accuracy (99.89%) and MCC (0.99) values. Similarly, out of 4,400 protein fragments of the blind dataset, HMM module c [file pone.0093907.s001.docx]

**Table S1:** Performance of SVM modules on genomic dataset using Amino acid composition as input (Learning parameters: t 2 g 0.01 c 6).

| Threshold | Sensitivity | Specificity | Accuracy | MCC |
| --- | --- | --- | --- | --- |
| -1 | 89.17 | 74.28 | 77.57 | 0.54 |
| -0.9 | 87.26 | 78.41 | 80.37 | 0.57 |
| -0.8 | 85.11 | 81.68 | 82.44 | 0.59 |
| **-0.7** | **82.65** | **84.34** | **83.96** | **0.60** |
| -0.6 | 80.31 | 87.09 | 85.59 | 0.62 |
| -0.5 | 77.60 | 89.26 | 86.68 | 0.64 |
| -0.4 | 75.14 | 91.06 | 87.53 | 0.65 |
| -0.3 | 72.31 | 92.32 | 87.89 | 0.65 |
| ***-0.2*** | ***69.60*** | ***93.65*** | ***88.32*** | ***0.65*** |
| -0.1 | 66.46 | 94.66 | 88.42 | 0.65 |
| 0 | 63.20 | 95.54 | 88.38 | 0.64 |
| 0.1 | 60.86 | 96.22 | 88.39 | 0.64 |
| 0.2 | 58.77 | 96.99 | 88.53 | 0.64 |
| 0.3 | 55.26 | 97.48 | 88.13 | 0.63 |
| 0.4 | 52.37 | 97.88 | 87.81 | 0.62 |
| 0.5 | 49.35 | 98.34 | 87.49 | 0.60 |
| 0.6 | 46.89 | 98.53 | 87.10 | 0.59 |
| 0.7 | 44.06 | 98.88 | 86.74 | 0.58 |
| 0.8 | 40.80 | 99.11 | 86.20 | 0.56 |
| 0.9 | 38.28 | 99.20 | 85.71 | 0.54 |
| 1 | 35.45 | 99.27 | 85.14 | 0.52 |

The point where sensitivity and specificity is roughly equal is highlighted in bold. The point of maximum MCC is highlighted in bold and italics.

**Table S2:** Performance of SVM module on the metagenomic datasets using amino acid composition as input (parameters for set B : t 2 g 0.002 c 6 and for set A - t 2 g 0.01 c 11).

| Threshold | Sensitivity | | Specificity | | Accuracy | | MCC | |
| --- | --- | --- | --- | --- | --- | --- | --- | --- |
|  | **A** | **B** | **A** | **B** | **A** | **B** | **A** | **B** |
| -1 | 79.57 | 96.50 | 71.95 | 22.28 | 73.91 | 41.29 | 0.46 | 0.22 |
| -0.9 | 74.55 | 94.79 | 78.21 | 30.93 | 77.27 | 47.29 | 0.48 | 0.26 |
| -0.8 | 69.69 | 92.47 | 83.02 | 40.46 | 79.59 | 53.78 | 0.50 | 0.31 |
| -0.7 | 65.36 | 89.64 | 86.53 | 50.46 | 81.08 | 60.50 | 0.51 | 0.36 |
| -0.6 | 61.25 | 86.06 | 89.35 | 60.37 | 82.12 | 66.95 | 0.52 | 0.41 |
| -0.5 | 57.43 | 81.63 | 91.4 | 69.94 | 82.66 | 72.93 | 0.52 | 0.45 |
| -0.4 | 53.64 | 76.32 | 93.06 | 78.38 | 82.92 | 77.85 | 0.52 | 0.50 |
| **-0.3** | **50** | 69.49 | **94.37** | 85.17 | **82.95** | 81.15 | **0.52** | 0.53 |
| -0.2 | 46.79 | 61.92 | 95.44 | 89.95 | 82.92 | 82.77 | 0.51 | 0.54 |
| **-0.1** | 43.61 | **54.80** | 96.24 | **93.33** | 82.69 | **83.46** | 0.50 | **0.54** |
| 0 | 40.81 | 47.55 | 96.98 | 95.52 | 82.52 | 83.23 | 0.50 | 0.52 |
| 0.1 | 38.42 | 40.94 | 97.49 | 96.98 | 82.29 | 82.62 | 0.49 | 0.50 |
| 0.2 | 36.09 | 34.82 | 97.95 | 98.03 | 82.03 | 81.83 | 0.48 | 0.47 |
| 0.3 | 33.89 | 29.17 | 98.26 | 98.70 | 81.70 | 80.89 | 0.47 | 0.44 |
| 0.4 | 31.85 | 23.81 | 98.52 | 99.15 | 81.36 | 79.85 | 0.46 | 0.40 |
| 0.5 | 29.81 | 19.27 | 98.79 | 99.47 | 81.04 | 78.93 | 0.45 | 0.36 |
| 0.6 | 27.87 | 14.93 | 99.04 | 99.66 | 80.72 | 77.95 | 0.44 | 0.32 |
| 0.7 | 26.25 | 11.48 | 99.18 | 99.78 | 80.41 | 77.16 | 0.43 | 0.28 |
| 0.8 | 24.76 | 8.29 | 99.32 | 99.87 | 80.13 | 76.41 | 0.42 | 0.24 |
| 0.9 | 23.28 | 5.83 | 99.43 | 99.93 | 79.83 | 75.82 | 0.41 | 0.20 |
| 1 | 21.81 | 3.57 | 99.49 | 99.96 | 79.50 | 75.26 | 0.39 | 0.16 |

The point of maximum MCC is highlighted in bold and in Red color for Set A. The point of maximum MCC is highlighted in bold and in Blue color for Set B.

**Table S3.** Performance of HMM modules on main and blind genomic dataset

| Set | Total number of proteins | Correctly predicted | Wrongly predicted | Shared domains | No hits found |
| --- | --- | --- | --- | --- | --- |
| Main  Positive | 1,625 | 1,153 | 14 | 330 | 128 |
| Main Negative | 5,715 | 5,375 | 30 | 274 | 36 |
| Blind  Positive | 100 | 72 | 3 | 17 | 8 |
| Blind Negative | 100 | 98 | 0 | 1 | 1 |

The detailed data for the predictions made by HMM module are provided in the above table. Out of 7,340 proteins of the main dataset, HMM module could classify 89.5% of the proteins with high Sensitivity (98.80%), Specificity (99.44%), Accuracy (99.33%) and MCC (0.98) values. Similarly, out of 200 proteins of the blind dataset, HMM module could classify 86.5% of the proteins with an accuracy of 98.26%.

**Table S4.** Performance of HMM modules on metagenomic Set A

| Set | Total number of proteins | Correctly predicted | Wrongly predicted | Shared domains | No hits found |
| --- | --- | --- | --- | --- | --- |
| Main  Positive | 12,538 | 4,920 | 37 | 2,688 | 4,893 |
| Main  Negative | 36,177 | 24,580 | 55 | 3,263 | 8,279 |
| Blind  Positive | 1,437 | 566 | 15 | 219 | 637 |
| Blind  Negative | 1,167 | 777 | 3 | 163 | 224 |

The detailed data for the predictions made by HMM module is provided in the above table. Out of 48,715 protein fragments of the main dataset, HMM module could classify 60.75% of the proteins with high Sensitivity (99.25%), Specificity (99.78%), Accuracy (99.69%) and MCC (0.99) values. Similarly, out of 2,604 protein fragments of the blind dataset, HMM module could classify 52.60% of the protein fragments with an accuracy of 98.68%.

**Table S5.** Performance of HMM modules on metagenomic Set B

| Set | Total number of proteins | Correctly predicted | Wrongly predicted | Shared domains | No hits found |
| --- | --- | --- | --- | --- | --- |
| Main  Positive | 21,458 | 5,533 | 18 | 2,736 | 13,171 |
| Main Negative | 62,303 | 29,111 | 19 | 3,459 | 29,714 |
| Blind  Positive | 2,426 | 641 | 9 | 229 | 1,547 |
| Blind Negative | 1,974 | 918 | 0 | 179 | 877 |

The detailed data for the predictions made by HMM module is provided in the above table. Out of 83,761 protein fragments of the main dataset, HMM module could classify 41.40% of the proteins with high Sensitivity (99.68%), Specificity (99.93%), Accuracy (99.89%) and MCC (0.99) values. Similarly, out of 4,400 protein fragments of the blind dataset, HMM module could classify 35.64% of the protein fragments with an accuracy of 89.24%.

**Table S6.** Comparison of the performance of SVM-HMM Hybrid approach with HMM and SVM modules

| **DATA** | | **HMM** | | **SVM** | | **HYBRID** | | |
| --- | --- | --- | --- | --- | --- | --- | --- | --- |
|  | | **Correct** | **Incorrect** | **Correct** | **Incorrect** | **Correct** | **Incorrect** | |
| **Blind**  **Genomic** | **P**  **(100)** | 72 | 3 | 84 | 16 | 92 | 8 | |
|  | **N**  **(100)** | 98 | 0 | 92 | 8 | 100 | 0 | |
| **Blind A** | **P**  **(1437)** | 566 | 15 | 1,047 | 390 | 1,184 | 253 | |
|  | **N**  **(1167)** | 777 | 3 | 1,101 | 66 | 1,142 | 25 | |
| **Blind B** | **P**  **(2426)** | 641 | 9 | 1,556 | 164 | 1,737 | 689 | |
|  | **N**  **(1974)** | 918 | 0 | 1,810 | 164 | 1,865 | 109 | |
| P – Positive dataset, N - Negative dataset. Default threshold for SVM module was -0.2 and default e-value of 1e-5 was used for HMM module.  Since, the generation of number of fragments depends on the length of the proteins, therefore, there are unequal number of fragments in the positive and negative datasets for Blind A and B.  In case of HMM, the number of correctly and incorrectly predicted proteins does not sum up to the total number because HMM does not make prediction on all the proteins. | | | | | | | |  |

**Table S7.** Comparison of time taken by MP3 and BLAST

| Number of Proteins | Time taken by BLAST  (in seconds) | Time taken by MP3  (in seconds) |
| --- | --- | --- |
| 100 | 15,007 | 7 |
| 500 | 71,494 | 35 |
| 1000 | 142,707 | 69 |
| 2000 | 285,486 | 144 |

**Table S8:** Results of MP3 on the three groups of proteins from the *Shigella flexineri* virulence plasmid

| Group | Protein | Function | Inhibition | MP3 prediction | Tag |
| --- | --- | --- | --- | --- | --- |
| Translocated | icsA | N-WASP recruiting protein |  | Pathogenic | S |
|  | icsB | Inhibits autophagy | Complete | Pathogenic | S |
|  | ipaA | Vinculin binding protein |  | Pathogenic | HS |
|  | IpaH1.4 | Putative E3 ubiquitin ligase |  | Pathogenic | HS |
|  | IpaH4.5 | Putative E3 ubiquitin ligase |  | Pathogenic | HS |
|  | IpaH7.8 | Putative E3 ubiquitin ligase |  | Pathogenic | HS |
|  | IpaH9.8 | E3 ubiquitin ligase |  | Pathogenic | S |
|  | IpgGB1 | G-protein mimic | Intermediate | Pathogenic | S |
|  | IpgGB2 | G-protein mimic | Complete | Pathogenic | S |
|  | IpgD | Inositol phosphate phosphatase | Complete | Pathogenic | S |
|  | OspB | Unknown | Weak | Pathogenic | S |
|  | OspC1 | Unknown | Intermediate | Pathogenic | S |
|  | OspD1 | Unknown |  | Pathogenic | S |
|  | OspD2 | Unknown |  | Pathogenic | S |
|  | OspD3 | Unknown | Intermediate | Nonpathogenic | S |
|  | OspE2 | CO-localizes with focal contacts |  | Pathogenic | S |
|  | OspF | MAPK phosphothreonine | Intermediate | Pathogenic | S |
|  | VirA | Microtubule-severing activity | Complete | Pathogenic | S |
| Non-translocated | acp | Periplasmic acid phosphatase |  | Nonpathogenic | S |
|  | ccdA | Antitoxin-plasmid segregation |  | Nonpathogenic | S |
|  | finO | Transfer of plasmids |  | Nonpathogenic | S |
|  | ipgA | TTSS chaperone |  | Pathogenic | S |
|  | ipgC | TTSS chaperone |  | Pathogenic | H |
|  | ipgE | TTSS chaperone |  | Pathogenic | H |
|  | ipgF | TTSS chaperone |  | Nonpathogenic | S |
|  | msbB2 | Lipid A modification |  | Nonpathogenic | HS |
|  | mvpA | Antitoxin-plasmid segregation |  | Nonpathogenic | S |
|  | mvpT | Toxin- plasmid segregation | Complete | Nonpathogenic | H |
|  | parA | Plasmid segregation | Intermediate | Nonpathogenic | HS |
|  | parB | Plasmid segregation |  | Nonpathogenic | HS |
|  | PhoN2 | Periplasmic acid phosphatase |  | Nonpathogenic | S |
|  | stbA | Plasmid segregation |  | Nonpathogenic | HS |
|  | stbB | Plasmid segregation |  | Pathogenic | S |
|  | traX | Transfer of plasmids |  | Pathogenic | S |
|  | trbH* | Unknown |  | - | - |
|  | ushA | Periplasmic UDP-sugar hydrolase |  | Nonpathogenic | H |
|  | virB | Transcriptional activator |  | Nonpathogenic | HS |
|  | virF | Transcriptional activator |  | Pathogenic | S |
| Group 3 | orf13* | - | - | - | - |
|  | orf212* | - | - | - | - |
|  | IpaJ | Unknown | Complete | Pathogenic | S |

***** The sequences for these proteins could not be found in the GenBank accession number provided in the work by Slogowski et al.

**Table S9.** Comparison of MP3 and VirulentPred on different test datasets

| Dataset | Method | Sensitivity | Specificity | Accuracy | MCC |
| --- | --- | --- | --- | --- | --- |
| Blind Dataset | **Virulent Pred** | 61.24 | 70.42 | 64.5 | 0.30 |
|  | **MP3** | 92 | 100 | 96 | 0.92 |
| Independent Dataset | **Virulent Pred** | **-** | **-** | 85% | **-** |
|  | **MP3** | **-** | **-** | 90% | **-** |
| Real Dataset (M. Tb.) | **Virulent Pred** | 81 | 34 | 57.5 | 0.16 |
|  | **MP3** | 97 | 97 | 97 | 0.94 |

The default threshold of -0.2 was used for SVM module and default e-value of 1e-5 was used for HMM module.

**Figure S1**: Comparison of performance of different kernels of SVM on genomic dataset shown by ROC plot.

The area under the ROC curve (AUC) for polynomial, RBF and linear kernel is 0.91, 0.91 and 0.86, respectively. Though, RBF and polynomial kernel have same AUC, however, the sensitivity value at zero threshold was higher in the polynomial kernel (76.12%) as compared to RBF kernel (73.39 %). Hence, polynomial kernel was selected for the prediction by SVM modules.

**Figure S2:** Comparison of performance of SVM modules using Amino Acid Composition (AAC) and dipeptide frequency as feature input for genomic dataset shown by ROC plot.

The Area under the ROC for SVM modules with dipeptide composition and amino acid composition are 0.91 and 0.90, respectively. The Area under the ROC curve is almost same in both the modules, however, the sensitivity value of AAC (63.20%) based module was much lower as compared to dipeptide composition based module (76.12%). Hence dipeptide composition modules were selected over AAC based modules.

**Figure S3:** Comparison of performance of SVM modules using Amino Acid Composition (AAC) and dipeptide frequency as feature input for metagenomic dataset A shown by ROC plot.

The performance of dipeptide composition based module was far much better as compared to AAC composition based module as apparent from the Figure. The area under the ROC curve for AAC module and dipeptide composition based module were 0.83 and 0.97 respectively. Hence, dipeptide composition was selected as feature input for the SVM modules constructed for metagenomic dataset A.

**Figure S4:** Performance comparison of SVM modules using Amino Acid Composition (AAC) and dipeptide frequency as feature input for metagenomic dataset B shown by ROC plot.

The performance of dipeptide composition based module was far much better as compared to AAC composition based module as apparent from the Figure. The area under the ROC curve for AAC module and dipeptide composition based module were 0.84 and 0.95 respectively. Hence, dipeptide composition was selected as feature input for the SVM modules constructed for metagenomic dataset B.

**Figure S5:** Performance comparison of different kernels of SVM on metagenomic dataset A (50 -100 aa) shown by ROC plot.

The area under the ROC curve for polynomial, RBF and linear kernel is 0.97, 0.97 and 0.80 respectively. In this case, polynomial and RBF kernels have similar performance for metagenomic dataset A. However, in all other cases, polynomial kernel showed better results, therefore, the polynomial kernel was selected as the default kernel for all the analysis using SVM.

**Figure S6:** Performance comparison of different kernels of SVM on metagenomic dataset B (30-50 aa) shown by ROC plot.

The area under the ROC curve for polynomial, RBF and linear kernel is 0.95, 0.91 and 0.75 respectively. As it is clearly seen the polynomial kernel is performing better then both the other kernels, hence, polynomial kernel was selected for the SVM modules constructed for metagenomic dataset B.

**Text S1.** The GI numbers of all the hypothetical proteins of the three pathogenic strains of *Mycobacterium* which were predicted as pathogenic by MP3 are given below.

*Mycobacterium tuberculosis str. Beijing NITR203:*

479312840, 479312842, 479312860, 479312862, 479312863, 479312865, 479312866, 479312882, 479312885, 479312896, 479312897, 479312900, 479312901, 479312938, 479312949, 479312961, 479312962, 479312970, 479312973, 479312989, 479312997, 479313036, 479313044, 479313045, 479313052, 479313056, 479313057, 479313080, 479313090, 479313117, 479313126, 479313131, 479313139, 479313140, 479313143, 479313145, 479313165, 479313170, 479313195, 479313212, 479313215, 479313219, 479313222, 479313235, 479313252, 479313266, 479313274, 479313283, 479313300, 479313310, 479313311, 479313318, 479313320, 479313329, 479313335, 479313337, 479313345, 479313360, 479313383, 479313395, 479313397, 479313402, 479313403, 479313412, 479313415, 479313418, 479313440, 479313446, 479313453, 479313467, 479313473, 479313486, 479313491, 479313492, 479313493, 479313497, 479313531, 479313537, 479313541, 479313551, 479313559, 479313565, 479313569, 479313573, 479313600, 479313601, 479313615, 479313621, 479313622, 479313626, 479313637, 479313655, 479313660, 479313663, 479313664, 479313665, 479313669, 479313673, 479313675, 479313680, 479313682, 479313685, 479313688, 479313696, 479313713, 479313715, 479313716, 479313758, 479313767, 479313788, 479313791, 479313792, 479313794, 479313795, 479313802, 479313831, 479313848, 479313851, 479313852, 479313876, 479313878, 479313879, 479313888, 479313889, 479313890, 479313894, 479313896, 479313930, 479313941, 479313949, 479313950, 479313958, 479313961, 479313962, 479313974, 479313983, 479313992, 479313995, 479314007, 479314009, 479314012, 479314014, 479314045, 479314055, 479314058, 479314059, 479314074, 479314086, 479314124, 479314129, 479314133, 479314136, 479314146, 479314171, 479314173, 479314176, 479314190, 479314192, 479314194, 479314228, 479314231, 479314258, 479314262, 479314270, 479314296, 479314320, 479314334, 479314343, 479314344, 479314354, 479314355, 479314361, 479314363, 479314387, 479314391, 479314392, 479314398, 479314399, 479314404, 479314405, 479314412, 479314413, 479314420, 479314427, 479314446, 479314480, 479314487, 479314502, 479314527, 479314530, 479314558, 479314585, 479314589, 479314607, 479314616, 479314630, 479314639, 479314643, 479314656, 479314667, 479314669, 479314681, 479314701, 479314710, 479314713, 479314720, 479314733, 479314738, 479314740, 479314749, 479314750, 479314755, 479314756, 479314774, 479314778, 479314788, 479314792, 479314796, 479314799, 479314802, 479314805, 479314813, 479314820, 479314832, 479314838, 479314864, 479314866, 479314880, 479314891, 479314892, 479314895, 479314897, 479314901, 479314902, 479314913, 479314937, 479314948, 479315000, 479315004, 479315007, 479315008, 479315009, 479315013, 479315017, 479315021, 479315027, 479315038, 479315039, 479315045, 479315067, 479315069, 479315076, 479315091, 479315092, 479315093, 479315102, 479315122, 479315123, 479315136, 479315153, 479315163, 479315179, 479315192, 479315195, 479315196, 479315226, 479315249, 479315277, 479315293, 479315294, 479315302, 479315314, 479315320, 479315355, 479315362, 479315369, 479315376, 479315383, 479315406, 479315436, 479315437, 479315439, 479315456, 479315459, 479315480, 479315493, 479315519, 479315527, 479315528, 479315544, 479315552, 479315556, 479315570, 479315600, 479315601, 479315613, 479315622, 479315652, 479315661, 479315673, 479315675, 479315676, 479315684, 479315699, 479315723, 479315724, 479315754, 479315760, 479315763, 479315768, 479315774, 479315780, 479315783, 479315801, 479315834, 479315853, 479315858, 479315866, 479315889, 479315922, 479315929, 479315933, 479315938, 479315973, 479315987, 479315996, 479316006, 479316017, 479316036, 479316039, 479316044, 479316045, 479316053, 479316062, 479316076, 479316091, 479316095, 479316097, 479316099, 479316100, 479316107, 479316142, 479316168, 479316169, 479316171, 479316173, 479316176, 479316177, 479316190, 479316193, 479316196, 479316209, 479316215, 479316231, 479316251, 479316252, 479316270, 479316302, 479316303, 479316326, 479316330, 479316338, 479316340, 479316346, 479316348, 479316349, 479316354, 479316357, 479316360, 479316361, 479316368, 479316389, 479316403, 479316407, 479316409, 479316419, 479316428, 479316435, 479316448, 479316478, 479316480, 479316488, 479316490, 479316507, 479316510, 479316511, 479316512, 479316516, 479316526, 479316530, 479316577, 479316582, 479316589, 479316592, 479316618, 479316623, 479316626, 479316632, 479316633, 479316635, 479316651, 479316656, 479316658, 479316661, 479316695, 479316697, 479316699, 479316701, 479316715, 479316724, 479316738, 479316744, 479316778, 479316781, 479316782, 479316809, 479316816, 479316821, 479316825, 479316834, 479316838, 479316846, 479316847, 479316850, 479316858, 479316859, 479316865, 479316869, 479316881, 479316882, 479316886, 479316889, 479316897, 479316898, 479316899, 479316903, 479316911, 479316912, 479316913, 479316915, 479316917, 479316920, 479316921, 479316923, 479316925, 479316929, 479316932.

*Mycobacterium leprae TN:*

15826872, 15826873, 15826890, 15826892, 15826896, 15826897, 15826899, 15826900, 15826904, 15826906, 15826915, 15826916, 15826922, 15826926, 15826931, 15826934, 15826949, 15826954, 15826968, 15826975, 15826980, 15826981, 15826984, 15826992, 15827000, 15827004, 15827008, 15827018, 15827025, 15827036, 15827059, 15827062, 15827065, 15827112, 15827124, 15827125, 15827134, 15827137, 15827138, 15827139, 15827149, 15827152, 15827172, 15827187, 15827188, 15827217, 15827218, 15827223, 15827224, 15827225, 15827229, 15827234, 15827235, 15827237, 15827248, 15827253, 15827264, 15827267, 15827269, 15827270, 15827290, 15827307, 15827310, 15827328, 15827337, 15827346, 15827354, 15827356, 15827359, 15827361, 15827388, 15827395, 15827400, 15827407, 15827424, 15827427, 15827441, 15827443, 15827446, 15827447, 15827450, 15827451, 15827453, 15827456, 15827458, 15827459, 15827472, 15827477, 15827478, 15827480, 15827485, 15827488, 15827490, 15827491, 15827497, 15827500, 15827511, 15827512, 15827513, 15827514, 15827540, 15827546, 15827580, 15827584, 15827597, 15827598, 15827600, 15827601, 15827615, 15827630, 15827631, 15827634, 15827652, 15827657, 15827658, 15827663, 15827666, 15827695, 15827709, 15827733, 15827745, 15827786, 15827788, 15827792, 15827793, 15827802, 15827809, 15827812, 15827821, 15827822, 15827826, 15827867, 15827882, 15827888, 15827918, 15827920, 15827947, 15827948, 15827951, 15827953, 15827966, 15827969, 15827971, 15827975, 15827977, 15828029, 15828034, 15828042, 15828048, 15828078, 15828086, 15828095, 15828097, 15828108, 15828117, 15828150, 15828151, 15828152, 15828154, 15828163, 15828178, 15828179, 15828185, 15828194, 15828198, 15828199, 15828202, 15828209, 15828211, 15828216, 15828218, 15828224, 15828225, 15828232, 15828242, 15828245, 15828246, 15828253, 15828256, 15828260, 15828276, 15828280, 15828282, 15828285, 15828288, 15828294, 15828298, 15828325, 15828327, 15828346, 15828358, 15828359, 15828361, 15828366, 15828367, 15828370, 15828371, 15828380, 15828383, 15828384, 15828387, 15828388, 15828394, 15828395, 15828396, 15828398, 15828399, 15828400, 15828401, 15828402, 15828403, 15828405, 15828411, 15828413, 15828414, 15828415, 15828419, 15828421, 15828429, 15828436, 15828437, 15828440, 15828445, 15828459.

*Mycobacterium tuberculosis H37Rv*

397671806, 397671807, 397671809, 397671837, 397671840, 397671841, 397671927, 397671935, 397671970, 397671978, 397672046, 397672053, 397672131, 397672149, 397672156, 397672159, 397672172, 397672245, 397672247, 397672266, 397672282, 397672295, 397672419, 397672486, 397672534, 397672548, 397672555, 397672568, 397672599, 397672605, 397672615, 397672618, 397672621, 397672629, 397672646, 397672693, 397672724, 397672728, 397672730, 397672763, 397672781, 397672787, 397672789, 397672803, 397672815, 397672824, 397672825, 397672826, 397672832, 397672874, 397672888, 397672896, 397672901, 397672934, 397672945, 397672951, 397672954, 397672973, 397673012, 397673073, 397673130, 397673194, 397673195, 397673199, 561108341, 397673292, 397673329, 397673352, 397673353, 397673357, 397673369, 397673388, 397673426, 397673470, 397673473, 397673495, 397673525, 397673533, 397673537, 397673566, 397673593, 397673617, 397673659, 397673669, 397673688, 397673690, 397673701, 397673706, 397673707, 397673729, 397673739, 397673753, 397673755, 397673758, 397673774, 397673792, 397673817, 397673819, 397673846, 397673849, 397673899, 397673919, 397673954, 397673958, 397673962, 397673963, 397673995, 397674023, 397674074, 397674113, 561108349, 397674145, 397674146, 397674158, 397674173, 397674178, 397674201, 397674206, 397674236, 397674259, 397674311, 397674323, 397674340, 397674375, 397674384, 397674448, 397674475, 397674522, 397674544, 397674554, 397674569, 397674570, 397674571, 397674615, 397674639, 397674709, 397674715, 397674723, 397674741, 397674846, 397674848, 397674883, 397674919, 397674928, 397674971, 397674993, 397674998, 397675007, 397675025, 397675036, 397675046, 397675051, 397675054, 397675058, 397675094, 397675120, 397675129, 397675141, 397675143, 397675161, 397675167, 397675184, 397675283, 397675301, 397675308, 397675311, 397675312, 397675320, 397675343, 397675344, 397675366, 397675445, 397675521, 397675527, 561108369, 561108370, 397675566, 397675591, 397675599, 397675607, 397675619, 397675645, 397675664, 397675673, 397675676, 397675691, 397675726, 397675729, 561108376, 397675757, 397675772, 397675792, 397675793, 397675810, 397675825, 397675861, 397675862, 397675874.
